# Versatility of cervicofacial flap in management of cutaneous cheek defects post tumour excision: a report of two cases

**DOI:** 10.4314/ahs.v23i1.53

**Published:** 2023-03

**Authors:** Ayodele Ogunkeyede, Remi Solagbade, Adigun Lawal

**Affiliations:** Department of Surgery, University of Ilorin Teaching Hospital, Ilorin. Nigeria

**Keywords:** Cheek defect, cervicofacial flap, basal cell carcinoma, squamous cell carcinoma, wide local excision

## Abstract

Reconstructing a large cutaneous cheek defect post tumour excision poses a great challenge to the reconstructive surgeon. The surgical options are limited for a functional and aesthetically acceptable outcome. The microvascular free flap which is currently the gold standard is still not a common place in our practice in Nigeria. Cervicofacial flap, a single stage procedure, offers an excellent alternative as it can be done for patients who are not fit for prolonged anesthesia and can even be undertaken under local anesthesia.

We presented two cases of patients with cheek tumors who had wide local excision after histological diagnosis of Basal Cell Carcinoma and Basaloid Squamous Cell Carcinoma. Both defects were closed with cervicofacial flap under general anesthesia. The flaps survived with no loss.

Cheek defect reconstruction with cervicofacial flap is simple, reliable and with similar favourable aesthetic outcome when compared with free flap procedure. It should be an important part of a reconstructive surgeon armamentarium.

## Introduction

Reconstructing a large cutaneous cheek defect post tumour excision poses a great challenge to the reconstructive surgeon.^1^ The microvascular free tissue transfer techniques used for the repair of extensive cheek defects are not routine in developing countries like Nigeria. Free tissue transfer, where available may not be feasible for high surgical risk patients who may not be able to withstand prolonged anesthsia. Hence, such patients will benefit from locoregional flaps such as cervicofacial flap that can be done over a reduced operating time and considerable postoperative recovery time. This flap technique had gone through various modifications since its introduction by Esser et al. in 1918 2 The modifications are in relation to superficial musculo-aponeurotic system (SMAS) plane dissection whether superficial or deep to it 3. It has robust random blood supply and it is usually inferiorly based. The reporting of this flap in literature is scanty especially among dark skinned people of African race. Hence, we report two cases of patients that underwent modified cervicofacial flap for reconstruction of Zone 1 cheek defects^6^. This technique reduced operating time and post op recovery time. The flap is reliable and with good patients’ selection, the outcome in dark skinned individual is acceptable as highlighted in these two cases.

## Case Reports

### Case 1

**Figure 1:**
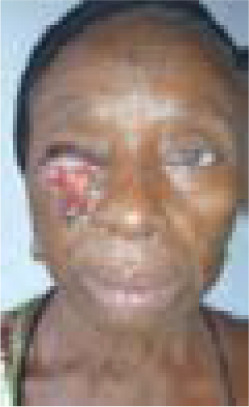


A 70-year-old woman, a petty trader, presented with long standing recurring right cheek ulcerated lesion with no known co-morbidities. She had a right cheek growth excised which became a chronic ulcer (Fig. 1). Initial specimen was not sent for histopathology. However, the lesion did not invade the underlying bone on assessment nor any evidence of metastasis. A presumptive diagnosis of basal cell carcinoma was entertained which was confirmed by histology. Hence, she was planned for wide local excision with modified cervicofacial flap for reconstruction. Postoperative period was uneventful as flap survived and she was discharged to clinic for follow up on postoperative day 10 (Fig 2). She was followed up for 10 months without recurrence before she was lost to follow up.

**Figure 2a:**
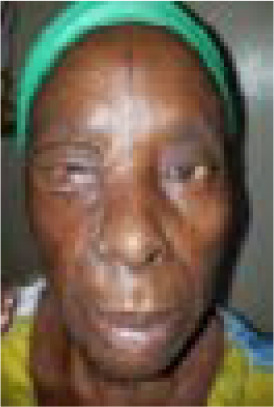

**Figure 2b:**
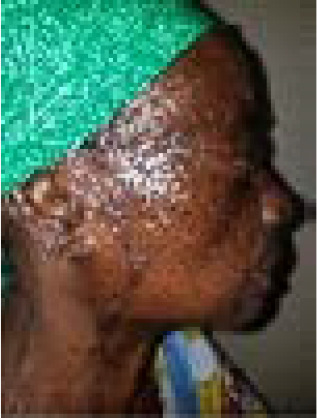

### Case – 2

**Figure 3:**
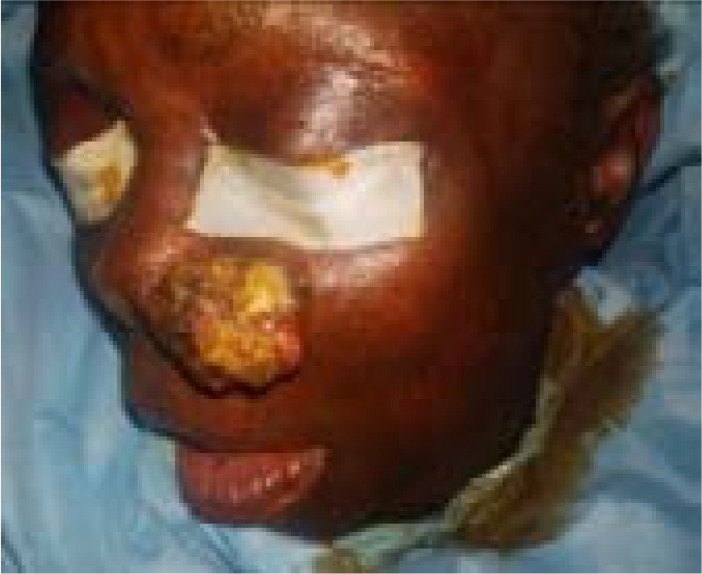


The second patient is a 65-year-old woman who presented with a left cheek ulcerated lesion involving the base of the left nasal alai of one year duration. On assessment, she did not have any significant co-morbities and the lesion did not invade the underlying bones (Fig. 3). The histopathological result was basaloid squamous cell carcinoma. She had a wide local excision plus reconstruction with a modified cervicofacial flap under general anesthesia. Flap survived and was discharged to clinic for follow up on postoperative day 11. She was followed up for 4 years without recurrence (Fig. 7a & b) Patient was satisfied with the cosmetic outcome.

**Figure 7a:**
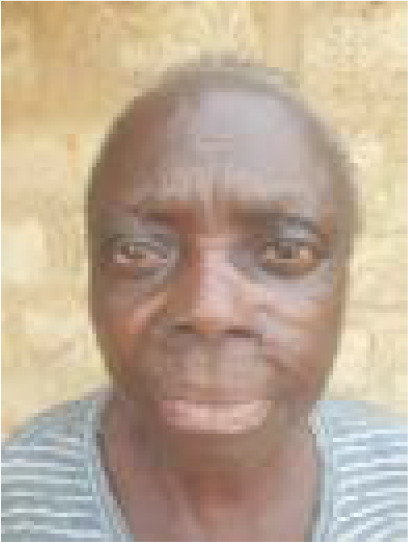


**Figure 7b:**
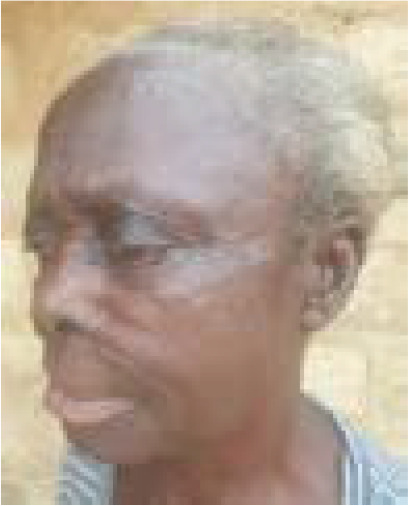


### Operative Procedure

**Figure 4:**
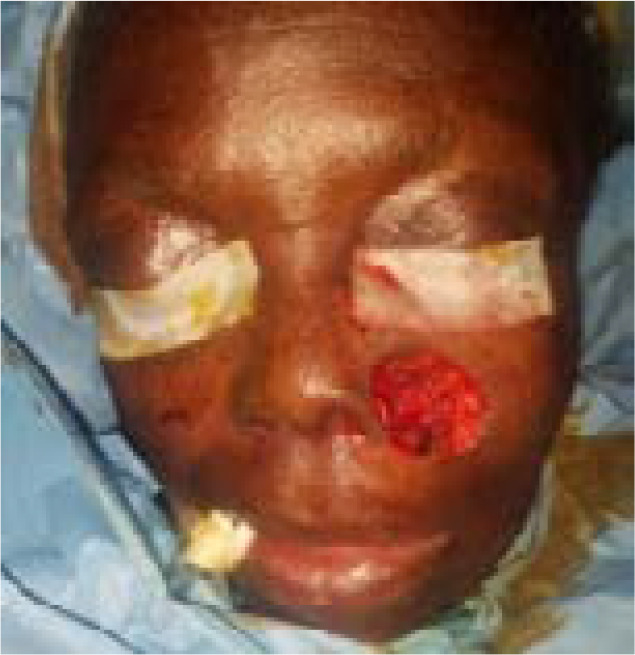


**Figure 5:**
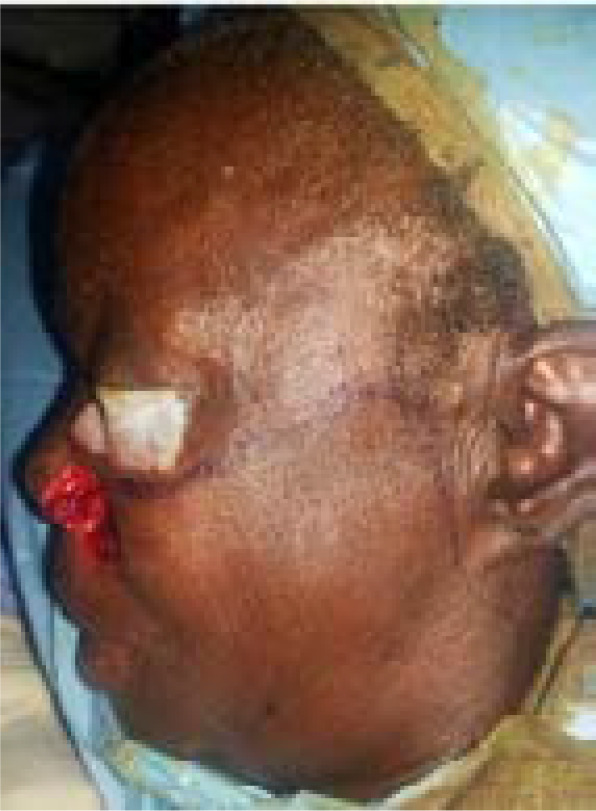


**Figure 6:**
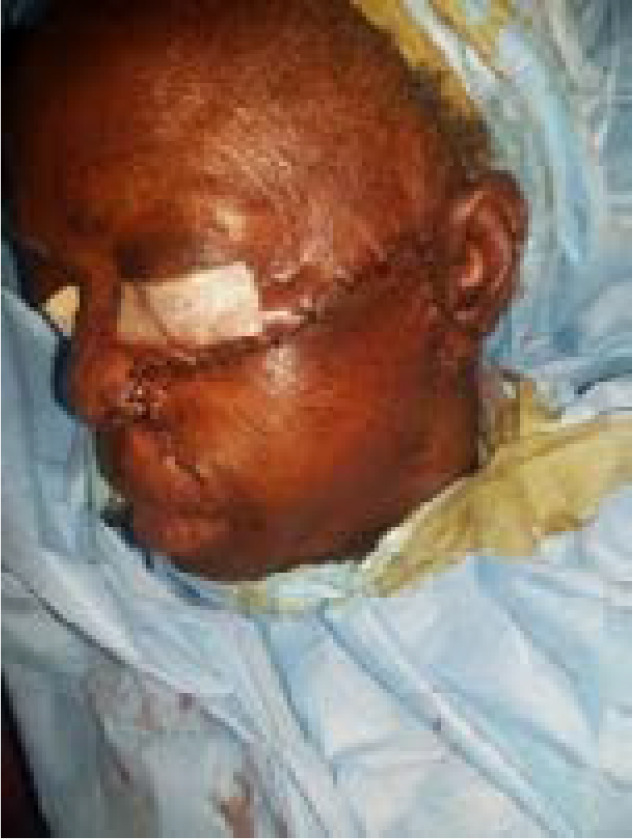


The patient was placed in a supine position with the head turned to the opposite side and with a head ring to stabilise the head and shoulder roll to extend the neck under general anaethesia. A 2% Lignocaine with adrenaline was injected in the subcutaneous plane after marking the 5 mm margin and along the proposed line of the incision to reduce blood loss and for post operative analgesia. The wide local excision of the lesions created Zone 1 cheek defects about 8cm x 6cm in case 1 and 4cm x 6cm in Case 2 (Fig. 4). The flaps were designed to camouflage the postoperative scars by placing them along the nasolabial fold, infraorbital imaginary line, extending it to the hairline following the preauricular crease to the posterior hair line and the neck to ensure that the flap covered the defects without resultant ectropion and aesthetic abnormality (Fig. 5). Both patients had supra-SMAS dissection for the cervicofacial flap. The flaps were closed in layers (Fig. 6). her postoperative period was uneventful as the flap survived without necrosis. There was no facial nerve deficit.

## Discussion

Reconstructing a large cheek defect especially those involving Zone 1 poses unique surgical challenge because of proximity to vital structures, scarcity of local flaps and possibility of ectropion as a complication. 4 The widely used classification of cheek defect by location is one described by Roth et al. 6 Zone 1 includes the infraorbital area and lower eye lid area. Although numerous options are available for closure of these defects based on size including primary closure, skin grafts, distant pedicled flaps, and free flaps, all of these techniques have their limitations. 1

The use of adjacent tissue in reconstruction of the cheek should be given a good consideration with respect to aesthetic and functionality. A simple primary closure could not be considered in our index cases because the defects were more than 4cm in sizes and attempting to close such in Zone 1 will lead to anatomic distortion and ectropion. Skin grafting which appears as a simple option is fraught with texture and colour mismatch and only reserve as an option when patient cannot undergo prolonged surgery due to severe co-morbidities. Camouflaging the scars of other pedicle flaps is usually very difficult. Free flaps are limited to patients that can undergo prolonged surgery and it requires expertise which is not readily available. Free flaps are usually bulky and require multiple stages for refining. Furthermore, free flaps do not provide colour and texture match. Modified cervicofacial flaps provide an excellent colour and texture match because it is adjacent to the defect, its s easier to elevate as a flap with good learning curve, less distortion of anatomic land mark, reduce operative time and surgical complications. Modified cervicofacial flaps can be carried out in patients with high surgical risk as it can be performed under local anaethesia with little or no donor site morbidity. The modified cervicofacial flaps was used by Kaplan in 1978 for coverage of defects following removal of cancers of the head and neck.^2^ This flap had undergone modifications based on the plane of dissection either superficial or deep to SMAS 3. The supra- SMAS flaps depend on the rich subdermal plexus of vessels, and the infra- SMAS flaps rely upon more reliable large perforator branches from facial and transverse facial arteries. The infra-SMAS flaps are believed to reduce post op complications including distal flap necrosis, epidermolysis because of its improved vascularity and provide thick skin flap. 8 However, we performed a supra-SMAS modified cervicofacial flaps for both of our patients which was much easier, with reduced operative time under general anaesthesia. 9 our patients did not have the potential complications of flap necrosis, lower eye lid ectropion nor epidermolysis. We were also able to avoid the potential facial nerve injury that could be associated with deep dissection technique.^2^,^10^ The scars were well camouflaged as we saw in the follow up after 4 years (Fig. 7a & b)

## Conclusion

The cervicofacial flap is a versatile, fast, simple and reliable technique for reconstructing a large cheek defect. It offers excellent aesthetic and functionally acceptable outcome comparable to that of a free flap technique. The free flap technique becomes more relevant in situation where volume loss of tissue is significant because it does not offer the colour match that local flap will provide. Utilizing the supra-SMAS dissection significantly reduces the risk of potential facial nerve injury and with good patient's selection; the scars are acceptable in dark skinned individuals.
